# Historic sequelae of lung tuberculosis

**DOI:** 10.11604/pamj.2018.30.210.16382

**Published:** 2018-07-16

**Authors:** Hanane Asri, Adil Zegmout

**Affiliations:** 1 Pulmonology Department, Mohammed V Military University Hospital, Rabat, Morocco, Faculty of Medicine and Pharmacy, Mohammed V University, Rabat, Morocco

**Keywords:** Fibrothorax, lung, tuberculosis

## Image in medicine

The patient is a 22-year-old woman who presented for the evaluation of chronic exertional dyspnea. Her past medical history was significant for tuberculosis lung five years ago. Lung auscultation revealed diminished respiratory sounds on the left hemithorax. Chest radiography (A) demonstrated shift of the mediastinal structures to the left with volume loss of the left lung and hyperlucency of the right lung. Pulmonary function tests demonstrated moderate restrictive lung disease. Computed tomography of the chest (B) revealed left bronchial stenosis with passive collapse of the left lung and attraction of the heart and mediastinal elements, compensatory emphysema of the right lung. Bronchoscopy showed obstruction of left main bronchus orifice with normal mucosa. The patient was diagnosed with calcified fibrothorax, believed to be secondary to chronic lung tuberculosis. She was managed conservatively, with no surgical intervention. Fibrothorax can be secondary to empyema or hemothorax, tuberculous pleurisy, asbestos exposure, collagen vascular isease and drug-induced pleuritis. Pleural fibrosis and thickening also may develop as a result of pleurodesis performed to therapeutically obliterate the pleural space treatment should be aimed at the underlying cause, with removal of offending agents if possible. Corticosteroids often are administered and surgical decortication is sometimes attempted; effectiveness of neither approach is established.

**Figure 1:**
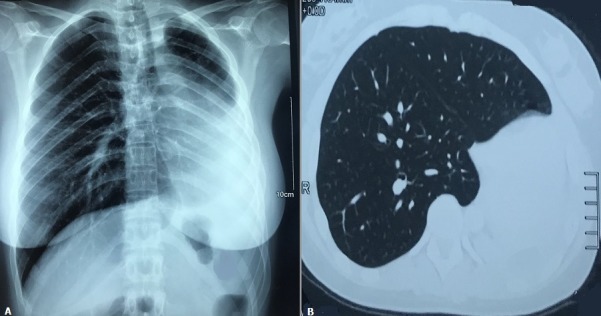
(A) chest radiography demonstrated shift of the mediastinal structures to the left with volume loss of the left lung and hyperlucency of the right lung; (B) computed tomography of the chest revealed left bronchial stenosis with passive collapse of the left lung and attraction of the heart and mediastinal elements, compensatory emphysema of the right lung

